# A Prebiotic Diet Containing Galactooligosaccharides and Polydextrose Attenuates Hypergravity-Induced Disruptions to the Microbiome in Female Mice

**DOI:** 10.3390/nu17152417

**Published:** 2025-07-24

**Authors:** Robert S. Thompson, Shelby Hopkins, Tel Kelley, Christopher G. Wilson, Michael J. Pecaut, Monika Fleshner

**Affiliations:** 1 Department of Integrative Physiology, University of Colorado Boulder, Boulder, CO 80301, USA; shho9335@colorado.edu (S.H.); tel.kelley@colorado.edu (T.K.); fleshner@colorado.edu (M.F.); 2 Center for Neuroscience, University of Colorado Boulder, Boulder, CO 80301, USA; 3 Center for Perinatal Biology, Loma Linda University, Loma Linda, CA 92318, USA; cgwilson@llu.edu; 4 Department of Basic Sciences, Loma Linda University, Loma Linda, CA 92318, USA; mpecaut@llu.edu

**Keywords:** microbiome, prebiotic, polydextrose (PDX), galactooligosaccharide (GOS), NLR ratio, RBCs, hypergravity

## Abstract

Background/Objectives: Environmental stressors, including spaceflight and altered gravity, can negatively affect the symbiotic relationship between the gut microbiome and host health. Dietary prebiotics, which alter components of the gut microbiome, show promise as an effective way to mitigate the negative impacts of stressor exposure. It remains unknown, however, if the stress-protective effects of consuming dietary prebiotics will extend to chronic altered-gravity exposure. Methods: Forty female C57BL/6 mice consumed either a control diet or a prebiotic diet containing galactooligosaccharides (GOS) and polydextrose (PDX) for 4 weeks, after which half of the mice were exposed to 3 times the gravitational force of Earth (3g) for an additional 4 weeks. Fecal microbiome samples were collected weekly for 8 weeks, sequenced, and analyzed using 16S rRNA gene sequencing. Terminal physiological endpoints, including immune and red blood cell characteristics, were collected at the end of the study. Results: The results demonstrate that dietary prebiotic consumption altered the gut microbial community structure through changes to β-diversity and multiple genera across time. In addition, consuming dietary prebiotics reduced the neutrophil-to-lymphocyte ratio (NLR) and increased red blood cell distribution width (RDW-CV). Importantly, the prebiotic diet prevented the impacts of altered-gravity on β-diversity and the bloom of problematic genera, such as *Clostridium_sensu_stricto_1* and *Turicibacter*. Furthermore, several prebiotic diet-induced genera-level changes were significantly associated with several host physiological changes induced by 3g exposure. Conclusions: These data demonstrate that the stress-protective potential of consuming dietary prebiotics extends to environmental stressors such as altered gravity, and, potentially, spaceflight.

## 1. Introduction

Stressor exposure, including altered gravitational conditions during spaceflight, can adversely affect the symbiotic relationship between host health and the gut microbiome [1,2,3,4,5,6,7]. Hypergravity and microgravity are examples of altered gravitational conditions experienced during spaceflight. Hypergravity occurs when the force of gravity exceeds the normal gravitational pull of Earth (1g) and is caused by a powerful thrust or deceleration; whereas microgravity refers to a gravitational environment that is significantly weaker than the Earth’s gravitational environment.

On Earth, it is currently impossible to expose any organism to a microgravity environment for more than a few minutes. Models simulating microgravity, such as hindlimb unloading, may not be suitable for examining systemic changes in brain–microbiome interactions or immune function. Therefore, alternative approaches are needed to capture the impacts of this space-relevant environmental factor. Although hypergravity and microgravity are distinct phenomena [8], both involve gravitational conditions that differ from those on Earth. Specifically, hypergravity at three times the normal gravitational pull of Earth (3g) can significantly disturb the gut microbiome of male mice [9], and 37 days of spaceflight can alter the gut microbiome of female mice [6]. It remains unknown, however, how 3g affects the female murine gut microbiome. Effective, efficient, and low-cost means of reducing the impacts of altered gravity, like those experienced before, during, and after spaceflight, are of interest.

Spaceflight and altered gravity negatively impact the gut microbiome, impairing the function of multiple leukocytes [10,11], altering red blood cell characteristics [12], and affecting pulmonary physiology [13]. Indeed, exposure to spaceflight and altered gravity have increased the neutrophil-to-lymphocyte (NLR) ratio [10], a clinical marker of systemic inflammation [14,15]. Others have reported that spaceflight can alter both hemoglobin levels and mean cell volume, although the dynamics of such changes vary by spaceflight duration [16,17]. The red blood cell distribution width (RDW) is a clinically meaningful readout to assess potential disease states where a higher value suggests more heterogeneity in the red blood cell size population and could indicate anemia [18]. RDW is minimally sex-dependent, but marginally higher after physical exercise, and typically higher in aged individuals [18]. Twelve days of spaceflight has been shown to alter several red blood cell characteristics including lowering RDW in female mice [19]. To date, less is known about how RDW is related to the microbiome. However, one report demonstrated that smokers have a lower RDW compared to non-smokers whilst simultaneously presenting with higher buccal microflora, suggesting a link [20]. In a recently published study, probiotic supplementation improved depressive symptoms but did not alter red blood cell characteristics [21]. These previous studies indicate that the stress of spaceflight and altered gravity disturb host physiology and microbial composition, and that these changes might be linked.

The stress of spaceflight and hypergravity can alter the gut microbiome and host physiological responses. One way to mitigate these stress-induced disruptions to the gut microbiome and host physiology is by consuming dietary prebiotics. Dietary prebiotics are primarily fermentable fibers that selectively stimulate the growth and activity of beneficial microorganisms and can contribute to gut health by modulating immune responses, metabolic disorders, and improving stressor responses. Dietary prebiotics, for example, can attenuate the adverse effects of various stressors, including acute traumatic stress [3], sleep deprivation [1], circadian disruption [2], and various psychosocial stressors [22,23]. Interestingly, dietary prebiotics, like galactooligosaccharide (GOS) and polydextrose (PDX), are known to both consistently change the gut microbial community [24] and alter red blood cell characteristics [25]. Thus, exploring whether administering dietary prebiotics can potentially mitigate some of the adverse effects of hypergravity exposure on the gut microbiome and host physiology is warranted. Compelling evidence suggests that exposure to environmental stressors associated with spaceflight alters the gut microbiome, and that ingestion of prebiotics is stress-protective; however, it remains unknown if the stress-protective effects of dietary prebiotics extend to hypergravity exposure specifically.

This study, therefore, tested if the ingestion of a diet enriched with the dietary prebiotics galactooligosaccharide (GOS) and polydextrose (PDX) could mitigate the 3g-induced changes to the female mouse gut microbiome and components of host physiology known to be altered during spaceflight. We hypothesize that the consumption of dietary prebiotics will (1) alter the female murine gut microbiome, and (2) attenuate the 3g-induced disruptions to the gut microbiome and host physiological responses in components of the immune system and red blood cell characteristics.

## 2. Methods

### 2.1. Animals

**Figure 1:**
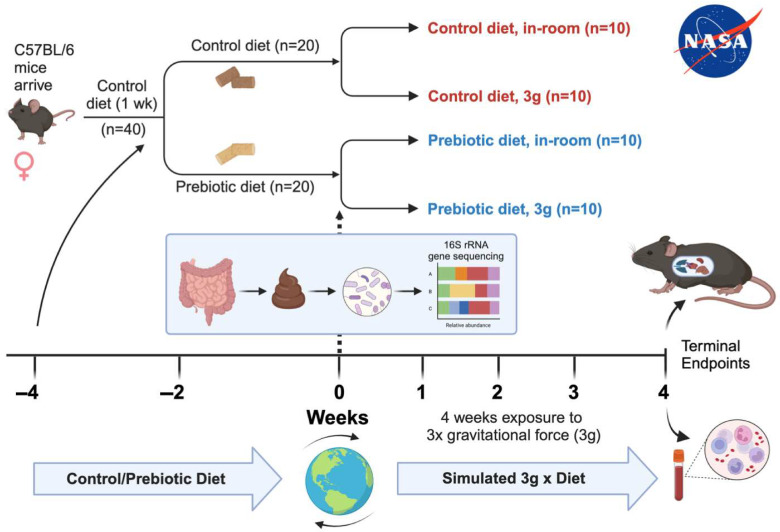
Timeline delineating experimental details including mouse species, diet types, durations of events, and terminal endpoints collected.

Female, 9-week-old C57BL/6 mice (10/group, 5 mice per cage, 40 mice total) were ordered from Jackson Laboratories and shipped to the NASA Ames Research Center Animal Care Facility (Moffett Field, CA, USA). Only female mice were studied because NASA primarily sends female mice into space due to total weight constraints during spaceflight. The temperature was maintained at an average recorded room temperature of 21 °C, while humidity was maintained between 30 and 50% throughout the experiment. The Institute for Animal Care Use Committee approved the protocol under Protocol Number: NAS-19-004-Y3. All mice were housed in disposable cages (Lab Products, LLC, Aberdeen, MD, USA) before and after transfer to the centrifuge facility. Mice received food/water ad libitum. Water was provided by HydroGel packs (NewCo Distributors, Inc., Cucamonga, CA, USA). Cages, food, and gel packs were changed twice per week. Weekly fecal pellets were also collected from a clean cage for each individual animal during cage changes to minimize disruption to the animals. At the end of the experiment (Figure 1), mice were euthanized via isoflurane overdose. Immediately after anesthesia euthanasia, blood was collected via cardiac puncture in EDTA-coated 1cc syringes.

### 2.2. Experimental Design

The female mice arrived, and all 40 mice were placed on a control diet for 1 week for acclimation to altitude and diet as shown in Figure 1 [24]. After 1-week acclimation, half of the mice were randomly placed on a prebiotic diet (*n* = 20), while the other half remained on the control diet (*n* = 20). All mice remained on their respective diets for the remainder of the study. After 4 weeks on either the control or prebiotic diet, half of the mice were exposed to centrifugation equivalent to three times that of earth’s gravity (3g), while the remaining mice were housed in the same room and cage types, but not exposed to 3g (in-room controls), which helped to control for noise stress and animal room differences. Mice were group housed with 5 animals per cage. The final number of animals per group was as follows: control diet, in-room (*n* = 10); control diet, 3g (*n* = 10); prebiotic diet, in-room (*n* = 10); prebiotic diet, 3g (*n* = 10). All mice were sacrificed at the end of the study and terminal fecal, organ, and blood samples were obtained (Figure 1).

### 2.3. NASA Centrifuge

The 1.22-m centrifuge is located in the Life Science Acceleration Research Facilities at NASA Ames Research Center, Moffett Field, CA, USA. It was uniquely built for hypergravity studies evaluating the effects of high g-forces on small model organisms ranging from microbes to plants and small animals. The 1.22-m Radius Centrifuge supports four specimen cabs, each adaptable to accommodate different types of experiments. Each of the specimen cabs can accommodate a variety of model organisms at various acceleration levels (10–45 RPM) during a single experiment reaching up to 4g. Powered habitat enclosures include continuous data and temperature monitoring. The four ground in-room control cabs, in addition to the four specimen cabs attached to the centrifuge, are located within the centrifuge room, while experiment operations are monitored and performed from an adjacent room.

### 2.4. Diets

All mice were fed either the control or prebiotic diets ad libitum. Both diets were calorically matched, and complete nutritional specifications of the diets have been previously published [2,3,4]. The prebiotic diet contained the following prebiotic substrates, which were absent from the control diet: galactooligosaccharides (GOS, 24.14 g/kg (7.0 g active); FrieslandCampina, Zwolle, The Netherlands), polydextrose (PDX, 7.69 g/kg (7.0 g active); Danisco, Terre Haute, IN, USA).

### 2.5. Fecal Sample Collection Procedures

The complete standardized protocols have been previously described in detail [2,26,27,28]. Briefly, each week, group-housed mice were transferred to individual clean cages to obtain subject-specific fecal samples. Upon defecation, samples were immediately stored in liquid nitrogen and frozen in a −80 °C freezer for later microbiome sequencing. Mice were then returned to their group housing and returned to their respective cabs.

### 2.6. 16S rRNA Gene Sequencing

Samples were same-day transferred from a −80 °C freezing in the Stress Physiology Lab (Department of Integrative Physiology, University of Colorado at Boulder, Boulder, CO, USA) on dry ice for less than 1 h to a −80 °C freezer in the Lozupone Lab (Department of Biomedical Informatics, University of Colorado Anschutz Medical Campus, Aurora, CO, USA) and processed for 16S rRNA sequencing. DNA was extracted from fecal samples, and the V4 region of the 16S rRNA gene was amplified using the 515f/806rB primer pair with the barcode on the forward read [29] and sequenced as previously described [30]. Samples were purified and precipitated to remove polymerase chain reaction (PCR) artifacts; samples were sequenced in multiplex on an Illumina HiSeq 2000. The sequencing data are publicly available at: https://qiita.ucsd.edu/study/description/16043 (accessed on 12 May 2025).

All target gene sequence processing was completed with Quantitative Insights Into Microbial Ecology (QIIME2). Raw sequencing data were trimmed and demultiplexed at 150 bases. Amplicon sequence variants (ASVs) were created using the deblur algorithm [31]. Using default parameters, Phylogeny was generated via SEPP [32] within the QIIME2 fragment insertion plugin. Taxonomy classification was completed via the QIIME2 feature classifier plugin [33] and based on Silva [34]. The resulting ASV table was filtered to remove mislabeled samples with a probability above 0.20 using the sample type field as described by [35,36]. The resulting table was then rarefied at 10,000 sequences/sample to correct for uneven sequencing depth due to amplification differences between samples.

Beta diversity was examined with a principal coordinate analysis (PCoA) using unweighted Unifrac distances (sensitive to rarer taxa) and weighted Unifrac distances (sensitive to abundances of taxa), which are the best ways to visualize the microbiome community as a whole [37]. For the analysis, PERMANOVA was used at each time point in QIIME2. Alpha diversity is a within-samples measure and was examined using evenness, observed features, and Faith’s Phylogenetic Diversity or Faith_PD [38]. Differential abundance was assessed on the ASVs using an analysis of the composition of microbiomes (ANCOM) [39] as implemented in QIIME2.

### 2.7. Hematology

Blood was collected and analyzed as previously described [12,19,40]. Briefly, mice were anesthetized with 3–4% isoflurane. Once unconscious and anesthetized (paw pinch), the chest cavity was opened and blood was collected in EDTA-containing 1cc syringes by cardiac puncture on wet ice, transferred to 1.7 mL microcentrifuge tube, and then evaluated on an Element HT5 Veterinary Hematology Analyzer (Heska/Antech, Loveland, CO, USA). Output variables include white blood cell (WBC) counts/percentages, neutrophils, lymphocytes, monocytes, eosinophils, and basophils. Red blood cells (RBC), hemoglobin concentration (HGB), hematocrit (HCT, percentage of whole blood consisting of RBC), and platelets (PLT) were also measured. Based on these parameters, standardized formulas were used to calculate the mean corpuscular volume (MCV, mean volume per RBC), mean corpuscular hemoglobin (MCH; mean weight of hemoglobin per RBC), mean corpuscular hemoglobin concentration (MCHC; concentration of hemoglobin per RBC), RBC distribution width (RDW; width of the RBC histogram produced by cell number × cell size), and the mean platelet volume (MPV; size of the average platelet).

### 2.8. Statistical Analysis

Data were analyzed using R statistics version 4.2.2 GUI 1.79 Big Sur ARM build (8160). Data depicted in the figures were made in Prism (version 10.1.1). For the gut microbiome analysis of Unifrac distance matrices, a permutation multivariate analysis of variance (PERMANOVA) was used at each time point [41,42]. Alpha diversity was analyzed using the Nonparametric Tests for Repeated Measures Data in Factorial Designs (nparLD) package, which was used for non-normally distributed data across time. To investigate differential abundances of genera level taxa between control and prebiotic diets, a first-level analysis of the composition of the microbiome (ANCOM) was performed on ASVs [39]. Once taxonomy was assigned based on ASVs, we performed a second level of analysis on genus level taxonomy assignments using the nparLD package as previously described [24]. All regression analyses were performed on log-transformed data when appropriate (Shapiro–Wilk) as previously described [2,4,24]. Tukey’s post hoc analysis was used when appropriate using the Nparcomp: Nonparametric relative contrasts effects (nparcomp) package for alpha diversity and relative abundances of the genera as previously described [24]. Results from all statistical ANOVA-Type tests for the relative abundance of specific genera are delineated in Table 1. Alpha was set at *p* < 0.05.

## 3. Results

### 3.1. Body/Food Weight–3g Reduced Body Weight and Food Consumption

**Figure 2:**
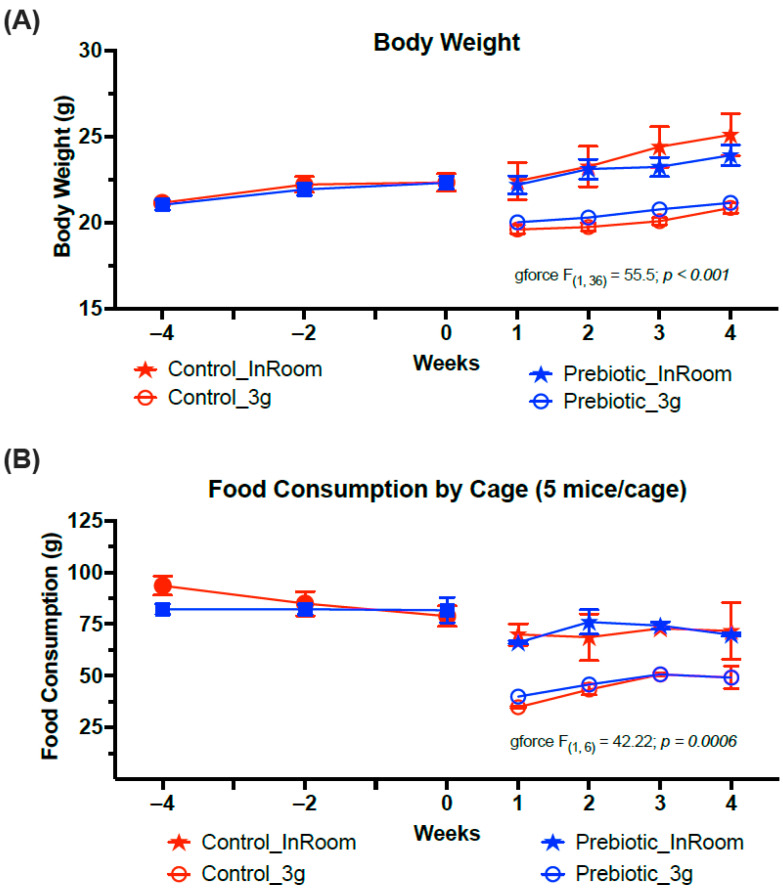
Exposure to 3g significantly reduced both (**A**) body weights and (**B**) food consumption. There were no effects of prebiotic diet on either food consumption or body weight and there were no interaction effects. The solid red circles represent control diet (*n* = 20), and solid blue squares represent prebiotic diet (*n* = 20) prior to 3g exposure.

Consumption of the prebiotic diet did not affect body weight when compared to control diet consumption across weeks of the experiment, consistent with previous studies. Exposure to 3g produced a significant body weight reduction (*F*_(1,36)_ = 55.5; *p* < 0.001, Figure 2A) and a significant reduction in food consumption regardless of diet (*F*_(1,6)_ = 42.22; *p* < 0.001, Figure 2B). There were no significant time-by-diet or time-by-3g interactions.

### 3.2. Microbiome

#### 3.2.1. β-Diversity–Prebiotic Diet Attenuated 3g-Induced Changes

**Figure 3:**
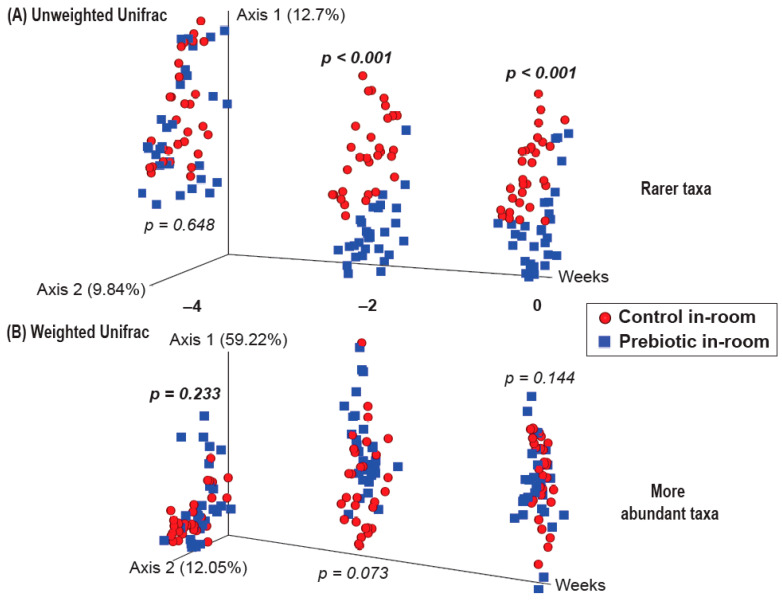
β-diversity PERMANOVA analysis showing both (**A**) Unweighted and (**B**) Weighted Unifrac from Weeks −4, −2, and 0 prior to the onset of 3g, with no significant differences between control and prebiotic diet groups at the start of the experiment (Week −4). Two weeks of consuming prebiotic diet was sufficient to alter Unweighted Unifrac (Week −2), which persisted until the onset of 3g exposure (Week 0). In contrast, prebiotic diet did not alter Weighted Unifrac at either time point.

**Figure 4:**
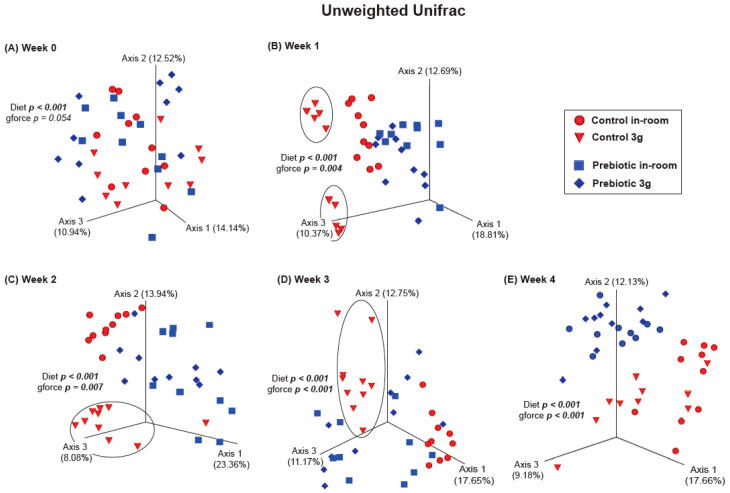
β-diversity PERMANOVA analysis showing Unweighted Unifrac (rarer taxa) at Week 0 before exposure to 3g and Weeks 1–4 during continuous exposure to 3g. (**A**) There were no significant effects of 3g exposure at Week 0, but during Weeks (**B**) 1, (**C**) 2, and (**D**) 3, the control diet group exposed to 3g was altered (circles) when compared with the other groups. By (**E**) Week 4, this effect, although still significant, appears to have waned, while the effect of consuming prebiotic diet became more pronounced.

**Figure 5:**
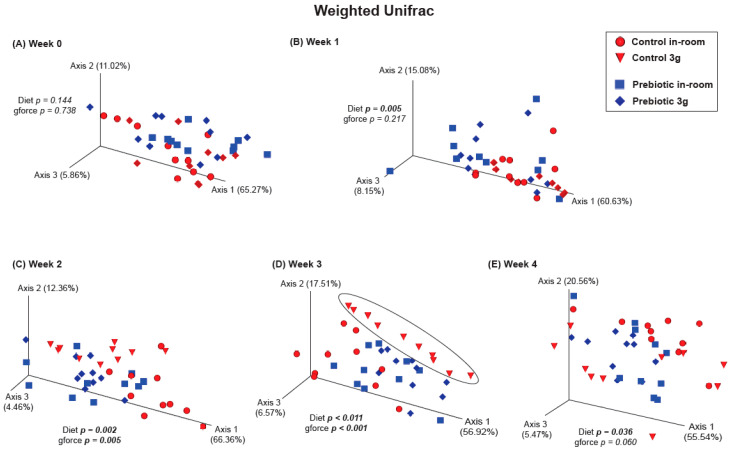
β-diversity PERMANOVA analysis showing Weighted Unifrac (more abundant taxa). (**A**) There were no significant effects of either prebiotic diet or 3g exposure at Week 0. (**B**) A significant effect of prebiotic diet on Weighted Unifrac emerged on experimental Week 1 (or 5 weeks total of consuming prebiotic diet), but there was still no effect of 3g exposure. Weighted Unifrac was significantly altered by both prebiotic diet and 3g exposure for Weeks (**C**) 2, and (**D**) 3, where, again, the control diet group exposed to 3g was altered (circle) when compared with the other groups. (**E**) Surprisingly, the prebiotic diet effect was less apparent, although still significant, by Week 4, while Weighted Unifrac failed to remain affected by continuous 3g exposure, thus revealing potential adaptations of higher abundance genera to chronic stress exposure.

There were no differences between the control and prebiotic diets in the community structure of the microbiome, or β-diversity, at the beginning of the experiment (Week −4) as measured by both Unweighted Unifrac (*pseudo*-*F*_(2,40)_ = 0.771; *p* = 0.648; Figure 3A) and Weighted Unifrac (*pseudo*-*F*_(2,40)_ = 1.37; *p* = 0.233; Figure 3B). By Week −2, there were significant differences between the control and prebiotic diets in Unweighted Unifrac (*pseudo*-*F*_(2,40)_ = 5.58; *p* < 0.001; Figure 3A) but not Weighted Unifrac (*pseudo*-*F*_(2,40)_ = 2.68; *p* = 0.073; Figure 3B). At Week 0, these prebiotic effects persisted for Unweighted Unifrac (*pseudo*-*F*_(2,40)_ = 4.95; *p* < 0.001; Figure 3A and Figure 4A), but there was still no effect on Weighted Unifrac (*pseudo-F*_(2,40)_ = 1.91; *p* = 0.144; Figure 3B and Figure 5A). The effect of a prebiotic diet on Unweighted Unifrac persisted throughout 3g exposure for Week 1 (*pseudo*-*F*_(2,40)_ = 4.68; *p* < 0.001; Figure 4B), Week 2 (*pseudo*-*F*_(2,40)_ = 4.98; *p* < 0.001; Figure 4C), Week 3 (*pseudo*-*F*_(2,40)_ = 4.38; *p* < 0.001; Figure 4D), and Week 4 (*pseudo*-*F*_(2,40)_ = 5.41; *p* < 0.001; Figure 4E). The prebiotic diet did alter Weighted Unifrac on Week 1 (*pseudo*-*F*_(2,40)_ = 6.41; *p* = 0.005; Figure 5B), Week 2 (*pseudo*-*F*_(2,40)_ = 9.04; *p* = 0.002; Figure 5C), and Week 3 (*pseudo*-*F*_(2,40)_ = 4.72; *p* = 0.011; Figure 5D), but this effect was less pronounced by Week 4 (*pseudo*-*F*_(2,40)_ = 3.47; *p* = 0.036; Figure 5E). Thus, it took 5 weeks of consuming a prebiotic diet to alter Weighted Unifrac (representing higher abundance genera) in female mice.

There were no effects of hypergravity, or 3g, during Week 0 in either Unweighted Unifrac (*pseudo*-*F*_(2,40)_ = 1.60; *p* = 0.054; Figure 4A) or Weighted Unifrac (*pseudo*-*F*_(2,40)_ = 0.368; *p* = 0.738; Figure 5A), although differences due to the prebiotic diet were already present as delineated above (Figure 3, Figure 4 and Figure 5; Weeks −4 to 0). By Week 1, Unweighted Unifrac was altered by 3g (*pseudo*-*F*_(2,40)_ = 2.62; *p* = 0.004; Figure 4B) but Weighed Unifrac was not (*pseudo*-*F*_(2,40)_ = 1.37; *p* = 0.217; Figure 5B). For the remaining weeks of the experiment, 3g altered β-diversity for both Unweighed Unifrac on Week 2 (*pseudo*-*F*_(2,40)_ = 2.62; *p* = 0.007; Figure 4C), Week 3 (*pseudo*-*F*_(2,40)_ = 4.19; *p* < 0.001; Figure 4D), and Week 4 (*pseudo*-*F*_(2,40)_ = 3.41; *p* < 0.001; Figure 4E). Weighed Unifrac was significantly altered on Week 2 (*pseudo*-*F*_(2,40)_ = 6.81; *p* = 0.005; Figure 5C), and Week 3 (*pseudo*-*F*_(2,40)_ = 8.41; *p* < 0.001; Figure 5D), but this effect waned on Week 4 (*pseudo*-*F*_(2,40)_ = 2.56; *p* = 0.060; Figure 5E).

#### 3.2.2. α-Diversity–Prebiotic Diet Reduced Measures of Alpha Diversity

**Figure 6:**
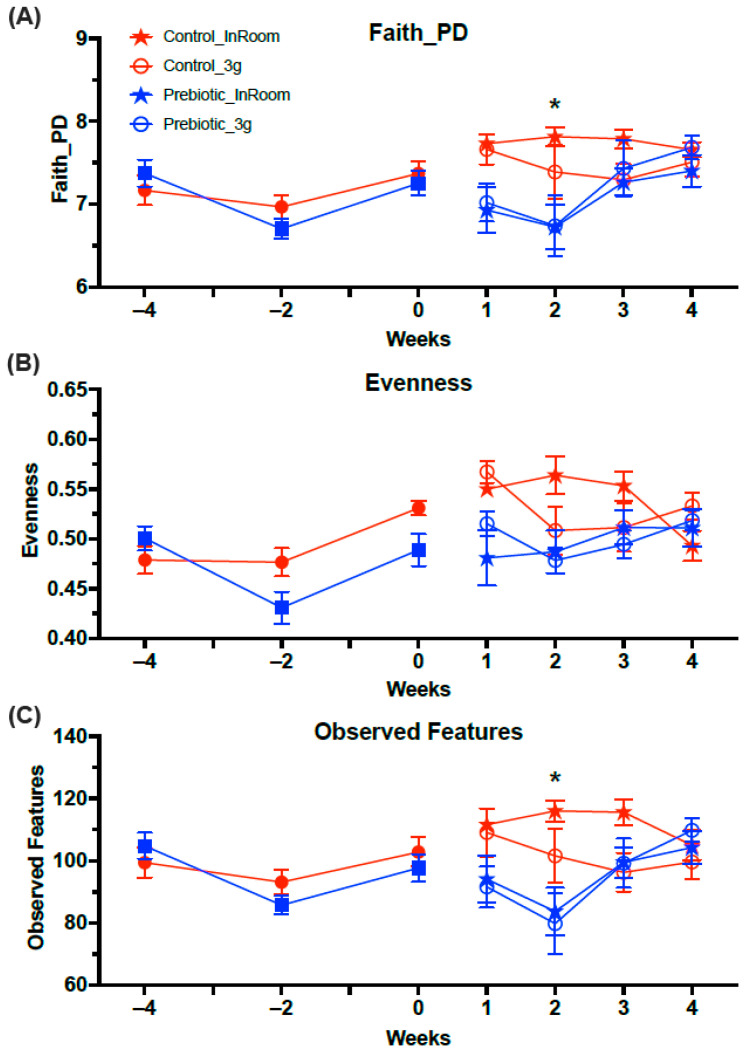
Alpha Diversity analyses across the experiment. (**A**) There was no effect of prebiotic diet on Faith_PD prior to 3g exposure, but mice eating prebiotic diet had significantly lower levels of Faith_PD in Week 2. (**B**) Evenness was significantly lower in the prebiotic diet groups both before and after exposure to 3g. Exposure to 3g also produced lower levels across time of Evenness in both diet groups. (**C**) The Observed Features were not different prior to 3g exposure, but there was a significant prebiotic diet effect in Week 5. The solid red circles represent control diet (*n* = 20) and solid blue squares represent prebiotic diet (*n* = 20) prior to 3g exposure. * *p* < 0.05 control, in-room vs. prebiotic, in-room.

The three measures of alpha diversity (Faith_PD, Evenness, and Observed Features) were lower overall in mice eating the prebiotic diet (Figure 6). Prior to 3g exposure, only Faith_PD had a three-way interaction between diet, 3g, and weeks (*F*_(1.85,32.90)_ = 3.53; *p* = 0.033; Figure 6A), while Evenness and Overserved Species were not different. In contrast, after 3g exposure, all measures of Alpha Diversity were lower in mice eating the prebiotic diet. There was a main effect of the prebiotic diet on Faith_PD (*F*_(1,22.12)_ = 11.78; *p* = 0.0006; Figure 6A), Evenness (*F*_(1,24.91)_ = 10.77; *p* = 0.001; Figure 6B), and Observed Features (*F*_(1,28.55)_ = 9.23; *p* = 0.002; Figure 6C), and there were diet-by-weeks effects for Faith_PD (*F*_(1,22.13)_ = 3.26; *p* = 0.022; see Figure 6A for results of post-hoc analysis), Evenness (*F*_(1,24.91)_ = 3.40; *p* = 0.021; Figure 6B), and Observed Features (*F*_(1,28.55)_ = 5.06; *p* = 0.002; see Figure 6C for results of post-hoc analysis). There was also a 3g-by-weeks effect on Evenness (*F*_(1,24.91)_ = 24.91; *p* = 0.009; Figure 6B) which was driven by lower Evenness in both diet groups from Week 1 to Week 2 of 3g exposure, but there were no significant post-hoc effects. Thus, it also took 5 weeks of consuming the prebiotic diet to significantly alter Faith_PD (Figure 6A) and the Observed Features (Figure 6C) in mice.

### 3.3. Taxonomy–Prebiotic Diet Attenuated 3g-Induced Increases in Several Taxa

**Figure 7:**
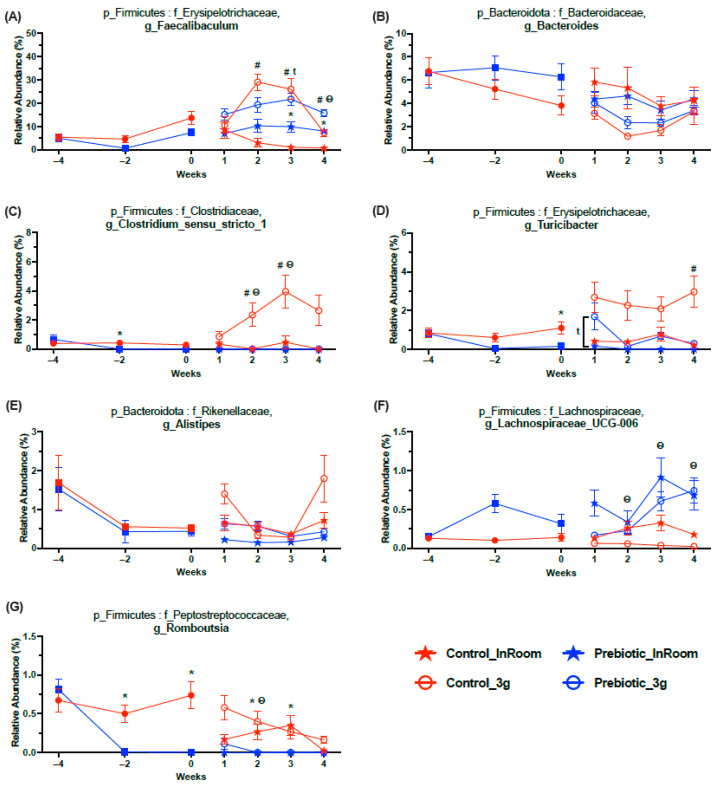
Relative abundance taxonomy data demonstrating the 7 genera most affected by 3g exposure (see Table 1 for statistics and figure for results of Tukey post-hoc analyses). (**A**) Exposure to 3g produced higher levels of *Faecalibaculum* in both diet groups primarily in Weeks 2 and 3. (**B**) In contrast, exposure to 3g produced lower levels of *Bacteroides* regardless of diet. (**C**) There was a protective effect of prebiotic diet on *Clostridium_sensu_stricto_1*, where this genus was higher in control diet mice exposed to 3g, an effect which was absent in prebiotic mice exposed to 3g. The genus also had somewhat lower levels in the prebiotic diet group (Week −2) prior to exposure to 3g. (**D**) Similarly, there was a protective effect of prebiotic diet on *Turicibacter*, where this genus was higher in control diet mice exposed to 3g, an effect which was attenuated, but not absent (Week 1), in prebiotic diet mice exposed to 3g. This genus also had lower levels in the prebiotic diet group (Week 0) prior to exposure to 3g. (**E**) The relative abundance of *Alistipes* was higher in mice eating control diet, and in all mice, exposed to 3g. (**F**) The relative abundance of *Lachnospiraceae_UCG-006* was higher in mice eating prebiotic diet and lower in mice exposed to 3g. Although the relative abundance of this genus was higher overall in mice eating prebiotic diet, 3g exposure produced lower levels in the control diet mice, while the levels of this genus in 3g-exposed mice eating prebiotic diet were less affected (Weeks 2, 3, and 4). (**G**) *Romboustia* was lower in mice eating prebiotic diet and 3g produced higher levels of this genus, primarily in the control diet 3g-exposed mice (Week 2). The solid red circles represent control diet (*n* = 20) and solid blue squares represent prebiotic diet (*n* = 20) prior to 3g exposure. * *p* < 0.05 control, in-room vs. prebiotic, in-room; **^#^** *p* < 0.05 control, in-room vs. control, 3g; ^t^ *p* < 0.05 prebiotic, in-room vs. prebiotic, 3g; ^θ^ *p* < 0.05 control, 3g vs. prebiotic, 3g.

There were seven higher abundance genera that were significantly affected by 3g (main effect), but they were also altered by the prebiotic diet (Figure 7A–G; statistics in Table 1). The high relative abundance genus *Faecalibaculum* was altered by diet across time and significantly impacted by 3g (main effect), such that *Faecalibaculum* levels were higher in both the control and prebiotic diet groups upon exposure to 3g (Figure 7A). The relative abundance of the genus *Bacteroides* was lower in mice exposed to 3g (main effect), but this genus was not altered by the prebiotic diet, although there was a trend prior to 3g exposure (Figure 7B). Of particular interest was *Clostridium_sensu_stricto_1*, which was altered by the prebiotic diet and much higher in the control diet, 3g group, thus revealing an attenuating effect of the prebiotic diet on this specific genus in response to 3g exposure (Figure 7C). Similarly, the genus *Turicibacter* was also altered by 3g exposure and the prebiotic diet. Upon initial exposure to 3g, both the control and prebiotic diet groups had higher levels of *Turicibacter*, but only in the control diet did the 3g group remain elevated throughout exposure to 3g, thus revealing another attenuating effect of the prebiotic diet on this genus (Figure 7D). *Alistipes* was also significantly altered by the prebiotic diet and 3g, where 3g produced higher levels (main effect) in this genus (Figure 7E; Table 1). The genus *Lachnospiraceae_UCG-006* had higher relative abundance in mice eating the prebiotic diet. However, 3g exposure led to lower levels of this genus in the control diet group, but not the prebiotic diet group, thus preventing a 3g-induced decrease in this genus (Figure 7F). While the 3g and prebiotic effects in this genus were significant, it should be noted that the difference in relative abundance denoted was only about 0.5%. In contrast, *Romboutsia* had higher relative abundance levels in mice eating the control diet, which remained higher in control diet mice exposed to 3g (Figure 7G).

**Figure 8:**
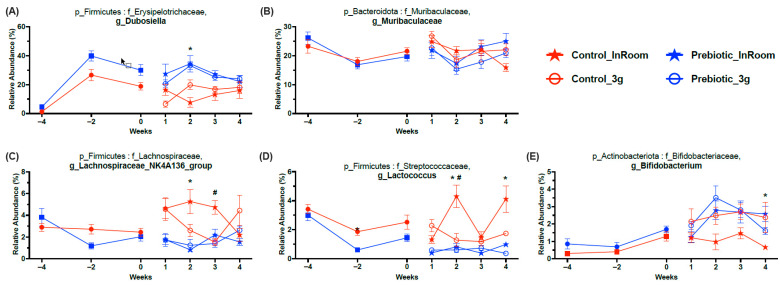
Relative abundance taxonomy data demonstrating 5 higher relative abundance genera significantly affected by prebiotic diet consumption and less affected by 3g exposure (see Table 1 for statistics and figure for results of Tukey post-hoc analyses). (**A**) Prebiotic diet consumption produced higher relative abundance levels in *Dubosiella*. (**B**) *Muribaculaceae* was slightly altered by prebiotic diet consumption, mostly varying across time, when compared to control diet. (**C**) The genus *Lachnospiraceae_NK4A136_group* had higher levels in the control diet groups, with 3g exposure producing lower levels in the control diet, 3g exposed group when compared to the control diet, in-room group. (**D**) *Lactococcus* also had higher levels in the control diet groups, with the control diet, in-room control diet group having the highest relative abundance levels compared to the other groups. (**E**) Finally, *Bifidobacterium* was higher in mice eating prebiotic diet when compared to mice eating control diet; however, 3g exposure produced dynamic changes as well. The solid red circles represent control diet (*n* = 20), and solid blue squares represent prebiotic diet (*n* = 20) prior to 3g exposure. * *p* < 0.05 control, in-room vs. prebiotic, in-room; **^#^** *p* < 0.05 control, in-room vs. control, 3g.

There were five higher abundance genera that were mainly affected by diet and less impacted by 3g (Figure 8; Table 1). The genus *Dubosiella* was the highest abundance genus increased by the prebiotic diet and also the genus most affected by the prebiotic diet. There was a small interaction between 3g and time in the *Dubosiella* genus, but no post-hoc effects (Figure 8A). *Muribaculaceae*, a prominent genus in mice, was slightly altered across time but only from Weeks 1 to 4, while 3g had no effect (Figure 8B). Both genera *Lachnospiracea_NK4A136_group* (Figure 8C) and *Lactococcus* (Figure 8D) had lower relative abundance levels in mice eating the prebiotic diet, while the 3g effects were less pronounced. Finally, the genus *Bifidobacterium* had higher relative abundance levels in mice eating the prebiotic diet; however, it took about 6 weeks of consuming the prebiotic diet for this effect to emerge (Figure 8E).

There were 26 more genera significantly altered by either the prebiotic diet or 3g; however, these were of much lower relative abundances when compared to those depicted in Figure 7 and Figure 8. In addition, the effects of both the prebiotic diet and 3g on these lower abundance genera were less consistent. These genera are graphed in Supplementary Figure S1 (0–2% relative abundance) and S2 (<0.5% relative abundance) and described in the Supplementary Materials with statistical results listed in Supplementary Table S1.

### 3.4. Organ Weights/Blood Panel Are Altered by Prebiotic Diet and 3g

**Figure 9:**
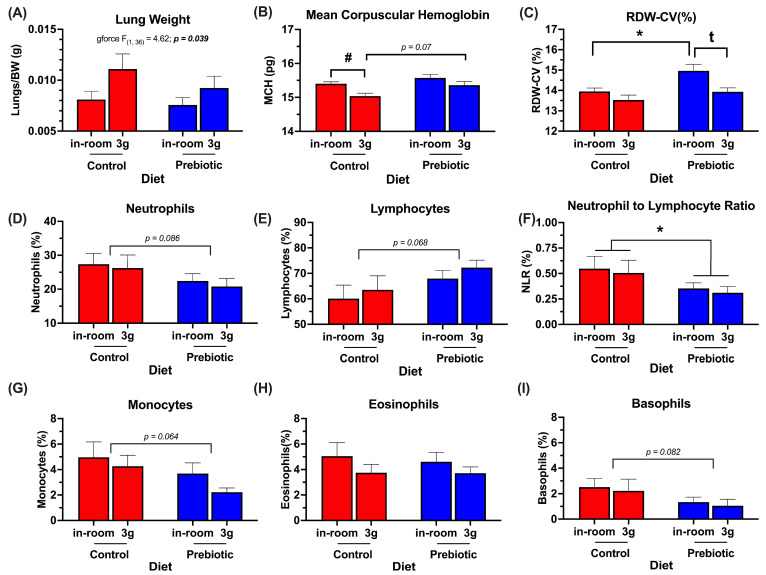
Significant terminal endpoint data (end of experimental Week 4) demonstrating main effects of prebiotic diet consumption and 3g exposure (see Table 2 for additional endpoint parameters that were not altered by either prebiotic diet or 3g, at the time measured). (**A**) Lung weights were higher in mice chronically exposed to 3g. (**B**) Dietary prebiotics modestly attenuated the 3g-induced decrease in mean corpuscular hemoglobin. (**C**) Mice eating prebiotic diet had higher levels of red blood cell distribution width, which was reversed upon exposure to 3g. All RDW-CV values measured were within the normal physiological range of 11–15%. There were trends towards prebiotic-induced decreases in (**D**) neutrophils, (**G**) monocytes, and (**I**) basophils, and a trend towards an increase in (**E**) lymphocytes. (**F**) There was a significant effect of dietary prebiotics lowering the neutrophil-to-lymphocyte ratio. (**H**) Eosinophils were unaffected by prebiotic diet. There were no effects of 3g exposure on the terminal white blood cell parameters measured. * *p* < 0.05 control, (in-room) vs. prebiotic, (in-room); **^#^** *p* < 0.05 control, in-room vs. control, 3g; ^t^ *p* < 0.05 prebiotic, in-room vs. prebiotic, 3g.

Table 2 denotes all terminal endpoints measured, including hematology. The significant results from Table 2 are graphed in Figure 9. There were no significant diet-by-3g interactions in these results; therefore, they are omitted from Table 2 for brevity. Lung weights (corrected for body weight) were significantly higher in mice exposed to 3g regardless of diet (*F*_(1,36)_ = 4.62; *p* = 0.039, Figure 9A). While this is somewhat dependent on the body weight correction, it is worth noting that the raw lung and mouse weights are not correlated (*p* = 0.737). In the hematology results, both the prebiotic diet (*F*_(1,36)_ = 7.76; *p* = 0.008, Figure 9B) and 3g (*F*_(1,36)_ = 10.47; *p* = 0.003, Figure 9B) altered the mean corpuscular hemoglobin (MCH). Specifically, exposure to 3g decreased MCH in the mice eating the control diet, but there was only a trend (*p* = 0.07) towards a protective effect of the prebiotic diet (see Figure 9B for results of Tukey post-hoc comparisons). The red blood cell distribution–coefficient of variation (RDW-CV) was also significantly increased by consumption of the prebiotic diet (*F_(_*_1,36)_ = 8.65; *p* = 0.006, Figure 9C), but this diet effect was reversed in mice exposed to 3g (*F*_(1,36)_ = 9.15; *p* = 0.005, see Figure 9C for results of Tukey post-hoc comparisons). The values measured for RDW-CV(%) were all within the normal range for RDW-CV of 11–15%. In mice eating the prebiotic diet, there were only trends towards lower values for neutrophils (*p* = 0.086; Figure 9D), monocytes (*p* = 0.064); Figure 9G), and basophils (*p* = 0.082; Figure 9I), while there was a trend towards an increase in lymphocytes (*p =* 0.068; Figure 9E). Interestingly, there was a small, but significant reduction in the neutrophil-to-lymphocyte (NLR) ratio in mice eating the prebiotic diet (*F_(_*_1,36)_ = 4.12; *p* = 0.049, Figure 9F) as well as the granulocyte-to-lymphocyte (GLR) ratio (F_(1,36)_ = 4.25; *p* = 0.047, Supplemental Figure S4), but 3g exposure had no effect.

### 3.5. Stepwise Multiple Regression Analysis Reveals Significant Relationships Between NLR, the Lungs, and the Gut Microbiome

We used a correlation network (Supplementary Figure S3A) and a correlation matrix (Supplementary Figure S3B) to investigate potential relationships between the higher abundance genera in Figure 7 and Figure 8 (Week 4 samples) and the significant terminal endpoint data in Figure 9. We found two independent significant stepwise multiple regression models.

**Figure 10:**
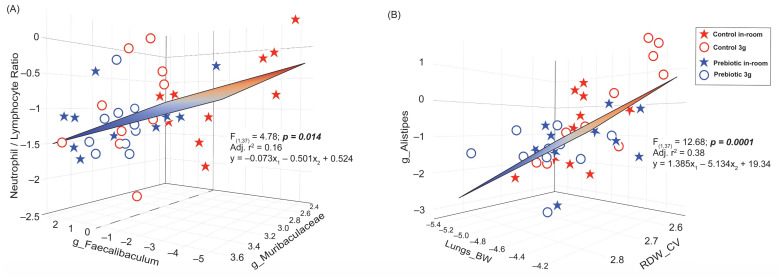
Multiple stepwise regression analyses demonstrating significant relationships between gut microbiome changes and blood cell changes altered by either prebiotic diet and/or 3g exposure. (**A**) A regression demonstrating a significant relationship between the NLR ratio and the two genera *Faecalibaculum* and *Muribaculaceae*. Mice eating a prebiotic diet, with a lower NLR ratio, tended to have higher levels of *Faecalibaculum* and higher levels of *Muribaculaceae*. This relationship is driven by mice with higher NLR ratios and lower levels of both *Faecalibaculum* and *Muribaculaceae*, like those eating control diet, not exposed to 3g (red stars). (**B**) A regression demonstrating a significant relationship between the genus *Alistipes*, lung weights, and the red blood cell distribution width. This relationship appears to be largely dependent on the 3g-exposed mice eating control diet (red circles), such that mice with higher levels of *Alistipes* had higher lung weights and lower red blood cell distribution widths.

Based on the correlation matrix, there was a significant two-factor stepwise multiple regression between the NLR, the genus *Faecalibaculum*, and the genus *Muribaculaceae* (*F*_(1,37)_ = 4.78; *p* = 0.014; Adj. r^2^ = 0.16; Figure 10A) which can be explained by the relationship: y = −0.073x_1_ − 0.501x_2_ + 0.524, where x_1_ = *Faecalibaculum* and x_2_ = *Muribaculaceae*. This relationship demonstrates that mice with a lower NLR (like those eating the prebiotic diet) tended to have higher levels of the genus *Faecalibaculum* (increased by 3g) and higher levels of *Muribaculaceae*. Based on both the correlation network and matrix, there was also a significant two-factor stepwise multiple regression between the genus *Alistipes*, the lung weight (corrected for body weight), and the RDW-CV (*F*_(1,37)_ = 12.68; *p* = 0.0001; Adj. r^2^ = 0.38; Figure 10B), where those mice with higher levels of the *Alistipes* had higher Lung_BW values but lower RDW-CV values and were described by the relationship: y = 1.385x_1_–5.134x_2_ + 19.34, where x_1_ = Lung_BW and x_2_ = RDW-CV. It is also worth noting here that the lung weight, whether corrected for body weight or not, was still significantly correlated with the genus *Alistipes* (*p* < 0.0001), but body weight was not (see Supplementary Figure S3B).

## 4. Discussion

These data demonstrate that consumption of dietary prebiotics and the stress of 3g exposure significantly alter the gut microbial community in female mice. Additionally, the prebiotic diet attenuated 3g-induced increases in *Clostridium_sensu_stricto_1* and *Turicibacter*. Exposure to 3g decreased body weight, food consumption, and MCH, but increased lung weight, while a prebiotic diet modestly decreased the NLR, and trended toward an attenuation of the 3g-induced decrease in MCH. Several significant relationships revealed that the prebiotic diet-induced dynamic changes in the gut microbial community and the alterations to both immune and red blood cell parameters were linked. These data further support that exposure to 3g can be detrimental to components of the host physiology, and that dietary prebiotics represent an effective, efficient, and low-cost means of mitigating some of the adverse effects of exposure to altered gravity, and possibly spaceflight, on host health.

The gut microbial community structure was significantly impacted by dietary prebiotics as evidenced by significant changes in both β-diversity (between groups) and α-diversity (within groups) results, which are consistent with previous studies [1,2,24]. Exposure to 3g had a more significant impact on altering β-diversity in mice eating a control diet when compared to those eating a prebiotic diet. While this effect was more apparent in lower abundance taxa (Unweighted Unifrac), it also appeared to be true, albeit temporarily, for more abundant taxa (Weighted Unifrac). These results are consistent with previous findings in male mice where 3g, but not 2g, altered β-diversity in the cecal community microbiome [9]. We replicated the observation that 3g altered β-diversity and reported that dietary prebiotics prevented this effect.

Consuming dietary prebiotics also altered α-diversity measures with the most considerable impact on evenness, similar to previous findings [1,24]. Exposure to 3g also appeared to alter evenness primarily in the control diet group, although there were no significant 3g post-hoc effects. Increases versus decreases in α-diversity are difficult to interpret meaningfully. However, it is consistently reported that dietary prebiotics [4,24] and stress exposure like 3g [9] and spaceflight [6] alter α-diversity, but these alterations are rarely directionally consistent. These findings and previous studies suggest that dietary prebiotics demonstrate stress-protective properties on the gut microbial structure as measured using both β-diversity and α-diversity.

Exposure to 3g significantly impacted several higher abundance genera. Exposure to 3g produced considerably higher relative abundance levels of *Faecalibaculum* (~10% higher), regardless of diet type. How 3g exposure affects *Faecalibaculum* has not previously been reported to the best of our knowledge. However, the oral administration of *Faecalibaculum* has been shown to induce depression-like phenotypes in male mice [43]. Higher levels of *Faecalibaculum* were also found in aged mice, which were associated with the pulmonary inflammatory response [44]. Our data and these previous studies support the notion that excessively high levels of *Faecalibaculum* may be maladaptive. In addition, excessively low levels of this genus are associated with early tumor growth [45]. Our data, in conjunction with these prior reports, support the idea that maintaining a consistent relative abundance level of the Faecalibaculum genus in the gut microbial community is paramount for maintaining both gut microbial community health and host health.

In contrast, exposure to 3g produced lower relative abundance levels of *Bacteroides* regardless of diet, albeit temporarily. Indeed, exposure to hindlimb unloading, a rodent model of microgravity, has previously been demonstrated to produce lower levels of several *Bacteroides* species in male mice [46]. *Bacteroides* appears to be stress-sensitive in general, since exposure to social disruption also led to lower levels of this genus [47]. Additionally, a high-fat diet also led to lower levels as well [48]. These examples indicate that stress-induced increases in *Faecalibaculum* combined with stress-induced reductions in *Bacteroides* may be especially problematic to maintaining balanced microbial community health, and, ultimately, host health.

A stress-protective effect of consuming dietary prebiotics was evident in two potentially harmful genera, *Clostridium_sensu_stricto_1* and *Turicibacter*. Exposure to 3g produced significantly higher levels of *Clostridium_sensu_stricto_1*, but only in the control diet group exposed to 3g. Similarly, the dietary consumption of GOS/PDX revealed a similar effect for *Turicibacter*, where 3g exposure led to higher relative abundance levels of *Turicibacter* in, primarily, the control diet group exposed to 3g. *Clostridium_sensu_stricto_1* was one of the top five dominant genera in colorectal cancer patients, and *Turicibacter* was higher in colorectal cancer patients when compared to control group patients [49], and higher levels of both genera were found in male rats with induced colitis [50,51]. Intriguingly, a defatted rice bran prebiotic attenuated the colitis-induced increase in both *Clostridium_sensu_stricto_1* and *Turicibacter* [50]. Finally, *Clostridium_sensu_stricto_1* has been shown to be increased in a chronic unpredictable mild stress model in male rats [52], while *Turicibacter* has recently been shown to be positively correlated with proinflammatory cytokines [53]. Taken together with our results, these findings suggest that both *Clostridium_sensu_stricto_1* and *Turicibacter* may be opportunistic bacteria, excessive increases of which disrupt gut microbial ecology and host health. We and others clearly show that dietary prebiotics can alleviate these maladaptive effects.

The effects of 3g exposure on *Alistipes*, *Lachnospiraceae_UCG-006*, and *Romboutsia* were less evident and less consistent across the experiment. The relative abundance levels of *Alistipes* were slightly lower in mice eating the prebiotic diet both before and during exposure to 3g. In comparison, a high-fat diet has been shown to lead to higher levels of *Alistipes* versus a control diet [48]. The relative abundance levels of *Alistipes* were higher in mice exposed to 3g, an effect that appeared to be driven mainly by the mice eating the control diet exposed to 3g. Indeed, altered gravity can produce higher relative abundance levels of *Alistipes* in human stool samples, although only the fecal samples were exposed to altered gravity [54]. Previous research has also demonstrated that *Alistipes* levels can be higher with stress exposure, and that this genus has been associated with psychosocial disorders like depression and anxiety [55].

*Lachnopsiraceae_UCG-006* had higher relative abundance levels in mice eating a prebiotic diet, while 3g exposure reduced levels of this genus, but only in mice eating a control diet exposed to 3g. Thus, dietary prebiotics, although resulting in higher overall relative abundance levels, prevented the 3g-induced decrease in *Lachnospiraceae_UCG-006* that was evident in the control diet 3g-exposed mice. A prebiotic diet-induced increase in *Lachnospiraceae_UCG-006* may benefit health, as it has been shown to have significant positive correlations with GABA and 5-HIAA [52]. Treatment with the flavonoid, procyanidin, led to higher relative abundance levels of *Lachnospiraceae_UCG-006* and was positively correlated with CD8 T-cells in lung metastatic female mice, suggesting a link between *Lachnospiraceae_UCG-006* and the immune response to C26 colon cancer cell metastasis [56]. The 3g-induced decreases in this genus were alleviated by prebiotic diet consumption.

The relative abundance levels of *Romboutsia* were much lower in mice eating a prebiotic diet. At the same time, exposure to 3g altered the relative abundance levels of the genera, but only in mice eating the control diet. While there are no reports on how spaceflight alters *Romboutsia*, pigs exposed to chronic social stress displayed elevated levels of *Romboutsia* [57]. Exposure to chronic mild stress was demonstrated to increase *Romboutsia* levels [58], while higher levels of *Romboutsia* were found in high-stress resilient mice [59]. It has also been shown that *Romboutsia* has been positively correlated with proinflammatory cytokines [53].

Our data, taken together with prior studies, suggest that the prebiotic diet induced effects on *Alistipes*, *Lachnospiraceae_UCG-006*, and *Romboutsia*, although somewhat variable, largely produced a stress-protective phenotype in the gut microbial community in response to 3g exposure.

There were five higher relative abundance genera mainly affected by consumption of the prebiotic diet, while the effect of 3g exposure was less prevalent. The relative abundance of *Dubosiella* was significantly higher in mice eating a prebiotic diet compared to mice eating a control diet. Increases in *Dubosiella* are likely beneficial given its potential role in anti-aging [60,61]. A recent study demonstrated a protective effect of the prebiotic inulin on the relative abundance levels of *Dubosiella* after the induction of experimental autoimmune encephalomyelitis; however, the effect of inulin alone on *Dubosiella* was not reported [62]. Decreases in relative abundance levels of *Dubosiella* have been found in sleep-deprived mice, which were improved by probiotic supplementation or fecal matter transplant [63], suggesting a link between sleep status and *Dubosiella*. Our previous work reports microbiome changes induced by GOS/PDX reduce the adverse effects of stressor exposure on sleep in male rats [1,2,3], and there is evidence that activity levels may also be linked with *Dubosiella* [60]. Based on the effects of dietary prebiotics on *Dubosiella* levels, as reported here and in the literature, future studies should examine whether prebiotic-induced increases in *Dubosiella* in female mice have any association with sleep/wake behavior, especially given the link between the gut microbiome and sleep disruptions [64,65].

Neither the prebiotic diet nor exposure to 3g altered the genus *Muribaculaceae* in our experiment. However, diet had a significant effect over time, mostly due to temporally dynamic changes in both diet groups. Data from the Jiang et al. (2019) study suggest that 37 days of spaceflight aboard the International Space Station may have produced somewhat lower relative abundance levels in the family *Muribaculaceae* in female mice [6]. One experiment examined the effects of hindlimb unloading (microgravity) on the microbiome and found reduced relative abundance levels of *Muribaculaceae* in male mice [46]. Curiously, Alauzet et al. (2019), examining the effects of 3g on the microbiome, did not detect the genus *Muribaculaceae* in the cecal samples of male mice [9]. Overall, it remains unclear if spaceflight and/or altered gravity reduces *Muribaculaceae*.

The remaining three genera, *Lachnospiraceae_NK4A136_group*, *Lactococcus*, and *Bifidobacterium* had much lower relative abundance levels when compared to *Dubosiella* and *Murbaculaceae*. Compared to the control diet group, mice eating dietary prebiotics had lower relative abundance levels of genera *Lachnospiraceae_NK4A136_group* and *Lactococcus* and higher relative abundance levels of *Bifidobacterium* when compared to the control diet group. The effects of 3g exposure on these three genera were minimal.

Although the overall impacts of 3g exposure on *Lachnospiraceae_NK4A136_group* were minimal in the control diet group, 3g may have temporarily reduced levels (time x 3g effect). This finding is consistent with the literature. Han et al. (2022) reported that altered gravity led to reduced relative abundance levels of *Lachnospiraceae_NK4A136_group* in human fecal samples [54]. *Lachnospiraceae_NK4A136_group* is generally considered to be a necessary short-chain fatty acid producing gut bacteria. Thus, large increases or decreases to the relative abundances of this genus may be detrimental to health. It has been reported that the relative abundance levels of *Lachnospiraceae_NK4A136_group* are (1) decreased by high-fat diet consumption in pregnant mice [66], (2) higher in diabetic mice [67], and (3) reduced in hypertensive patients with obstructive sleep apnea [68], while *Lachnospiraceae_NK4A136_group* is strongly associated with the common gastrointestinal disorder functional dyspepsia [69]. These studies suggest that the genus *Lachnospiraceae_NK4A136_group* is likely essential for maintaining host health, but that different physiological states may differentially benefit from increased or decreased relative abundances of *Lachnospiraceae_NK4A136_group*.

The effects of both dietary prebiotics and 3g exposure on *Lactococcus* were similar to *Lachnospiraceae_NK4A136_group* to some extent. The relative abundance levels of *Lactococcus* were lower in mice consuming dietary prebiotics, with minimal effects of 3g exposure. The species *Lactococcus lactic*, within the *Lactococcus* genus, is generally thought to be beneficial for host and mucosal health [70]; however, less is known about how 3g exposure affects this genus, and there are varying reports about the prebiotic effects. A recent study by Cheng et al. (2025) used an in-vitro fecal culture experiment to demonstrate that GOS supplementation led to lower relative abundance levels of *Lactococcus* when compared to the control culture [71], and those findings are consistent with what we report here; however, another study demonstrated a GOS-induced increase in *Lactococcus* in lactose intolerant patients [72].

Finally, *Bifidobacterium*, a well-accepted health-promoting probiotic, was significantly increased in mice eating a prebiotic diet compared to those eating a control diet, with minimal effects of 3g exposure. The taxonomic data presented here, taken together with previous studies, support the conclusion that the consumption of dietary prebiotics promotes a gut microbial community structure that is more resistant to stress-induced perturbations, which likely extends host health benefits.

How stressors experienced during spaceflight, including altered gravity, affect host pulmonary and immune responses remains a prominent area of interest [73]. The current data demonstrate that chronic exposure to 3g significantly elevated lung weights. It is possible that chronic hypergravity exposure induced slight damage to the lungs due to their delicate anatomy; however, dietary prebiotics did not attenuate this effect.

Exposure to 3g also altered two red blood cell characteristics. Specifically, 3g exposure reduced the mean corpuscular hemoglobin (MCH) in female mice, consistent with previous findings [12,74]. The MCH indicates the amount of hemoglobin in each red blood cell; thus, decreases in the MCH suggest less hemoglobin per red blood cell and may signify hypergravity-induced iron-deficient anemia, given that these were otherwise healthy mice. Indeed, space anemia is a condition observed in prolonged space flight [75,76]. Intriguingly, there was a trend (*p* = 0.07) towards a protective effect of the prebiotic diet for this response.

There were no other effects at the time of sample collection, of either the prebiotic diet or 3g exposure, on the remaining red blood cell characteristics, with the notable exception of red blood cell distribution width (RDW-CV). The consumption of dietary prebiotics increased RDW-CV in female mice; however, 3g exposure reversed this prebiotic diet-induced increase. Generally, a higher RDW-CV is associated with negative health outcomes [18]; however, it should be taken into account that the statistically elevated RDW-CV values in mice eating a prebiotic diet not exposed to 3g were still within the normal physiological range of 11–15%, thus not pathological. The elevated RDW-CV levels in mice eating a prebiotic diet were clearly reversed in mice exposed to 3g. There were no significant effects of 3g on RDW-CV in control diet mice. Taken together, these data imply that the adverse effects of 3g exposure on components of red blood cell characteristics may be protected, in part, by dietary prebiotic consumption.

If dietary prebiotics, which alter the gut microbiome community, are indeed stress protective, as has been demonstrated in the literature [1,2,3,23,76,77], then the 3g-induced alterations to host physiological responses and the 3g-induced changes to the gut microbiome should be linked. Indeed, a regression analysis indicated that the 3g-induced increase in lung weight and prebiotic diet-induced increase in RDW-CV were linked with the 3g-induced increase in the relative abundance levels of *Alistipes*. These findings add to a growing body of evidence that prebiotic diet-induced changes to the gut microbial community can benefit host health, particularly during stressor exposure.

In contrast with the effect of 3g exposure on red blood cell characteristics, 3g did not alter any white blood cell parameters in the blood at the time point collected. Dietary prebiotics, however, produced a slight but significant reduction in the neutrophil-to-lymphocyte (NLR) ratio, which is used as a clinical determinant of inflammation status [14,15]. Although a consensus does not yet exist, a higher NLR ratio has been associated with mortality, the severity of stroke, post-stroke complications, and MS patients in relapse [78,79,80,81,82]. The NLR has also been linked with coronary artery disease [83], multiple sclerosis [84], and colorectal cancer [85]. Clinically, a higher NLR appears to reflect a negative inflammatory state, which suggests that a lower NLR likely reflects an improved inflammatory state. Intriguingly, there is some evidence that prebiotics, specifically oligosaccharides, can reduce the NLR [86,87]. The data presented here demonstrate a small but significant reduction in the NLR ratio in female mice consuming the prebiotic diet regardless of exposure to 3g. These results are consistent with previous findings and suggest that dietary prebiotics may exert stress-protective effects through a reduced NLR ratio, indicative of decreased inflammatory status.

Intrigued by the lower NLR ratio induced by the prebiotic diet, we examined how changes to this parameter related to our prebiotic-induced gut microbiome changes. Our results demonstrated a significant relationship between the NLR ratio and two genera: *Faecalibaculum* (increased by 3g exposure) and *Muribaculaceae*. This relationship is consistent with a previous study demonstrating that a lower NLR was associated with a greater microbiome diversity [15]. Our previous work reports that dietary prebiotics are stress-protective [1,2,3,4,23,77]. Here, we present a significant relationship between a genus that is impacted by 3g exposure and the NLR ratio which is reduced (i.e., stress-protective) by prebiotic diet consumption. These results further strengthen the case that dietary prebiotics are stress-protective and suggest that their stress-protective effects extend, in part, to the adverse effects of prolonged exposure to hypergravity.

Although we identified significant relationships between bacterial genera and the impacts of 3g, regression analyses do not imply causation. Future research is needed to determine if changes in the associated genera directly induce stress-protective effects.

## 5. Conclusions

Our results closely align with previous findings and contribute to a growing body of literature demonstrating both the stress-protective and health-promoting effects of dietary prebiotic consumption. These data strongly support the conclusion that optimizing a stress-protective gut microbial community, both before and during spaceflight exploration, could mitigate the negative effects of stress exposure, including altered gravity, on the gut microbial community structure and host health.

## Figures and Tables

**Table 1:** Nonparametric Longitudinal Data (naprLD) demonstrating the effects of diet, 3g, time, and interaction effects. The effects of diet are denoted during Week −4 to Week 0 at the beginning of the experiment. After 3g started, the effects of both diet and 3g exposure are listed. Not significant (ns).

Nonparametric Longitudinal Data (nparLD) Table: ANOVA-Type Statistics (ATSs)								
Altered Mainly by 3g	Weeks −4–0 (Pre 3g)		Weeks 1–4 (Post 3g)					
	Diet	Diet × Time	Diet	Gforce (3g)	Diet × 3g	Diet × Time	3g × Time	Diet × 3g × Time
Faecalibaculum	ns	*F*_(2,29.87)_ = 3.36; *p* = 0.039	*F*_(1,2.11)_ = 10.94; *p* = 0.0009	*F*_(1,2.11)_ = 57.41; *p* = 3.54 × 10^−14^	*F*_(1,2.11)_ = 4.02; *p* = 0.045	ns	*F*_(3,26.70)_ = 6.27; *p* = 0.002	*F*_(3,26.70)_ = 4.62; *p* = 0.009
Bacteroides	*F*_(1,1.92)_ = 3.23; *p* = 0.072	ns	ns	F_(1,2.59)_ = 16.21; *p* = 0.00006	ns	ns	ns	ns
Clostridium_sensu_stricto_1	ns	*F*_(2,33.45)_ = 8.35; *p* = 0.0003	*F*_(1,2.96)_ = 23.12; *p* = 0.000002	*F*_(1,2.96)_ = 8.77; *p* = 0.003	*F*_(1,2.96)_ = 6.91; *p* = 0.009	ns	*F*_(3,20.14)_ = 3.41; *p* = 0.02	ns
Turicibacter	ns	*F*_(2,30.37)_ = 12.95; *p* = 0.000003	*F*_(1,2.54)_ = 22.12; *p* = 0.000003	*F*_(1,2.54)_ = 29.11; *p* = 6.83 × 10^−8^	ns	*F*_(3,20.82)_ = 3.96; *p* = 0.012	ns	*F*_(3,20.82)_ = 3.55; *p* = 0.019
Alistipes	*F*_(1,1.83)_ = 10.00; *p* = 0.0009	ns	*F*_(1,2.62)_ = 24.28; *p* = 8.32 × 10^−7^	*F*_(1,2.62)_ = 10.97; *p* = 0.0009	*F*_(1,2.62)_ = 10.29; *p* = 0.001	ns	ns	*F*_(3,27.49)_ = 3.31; *p* = 0.024
Lachnospiraceae_UCG-006	*F*_(1,1.97)_ = 23.59; *p* = 0.000001	ns	*F*_(1,2.74)_ = 46.94; *p* = 7.30 × 10^−12^	*F*_(1,2.74)_ = 8.95; *p* = 0.003	*F*_(1,2.74)_ = 7.10; *p* = 0.008	*F*_(3,21.76)_ = 17.45; *p* = 0.0006	ns	*F*_(3,21.76)_ = 2.97; *p* = 0.035
Romboutsia	*F*_(1,1.80)_ = 21.80; *p* = 0.000003	*F*_(2,28.48)_ = 20.12; *p* = 9.68 × 10^−9^	*F*_(1,2.87)_ = 100.80; *p* = 1.02 × 10^−23^	*F*_(1,2.87)_ = 4.46; *p* = 0.035	ns	ns	*F*_(3,22.64)_ = 3.42; *p* = 0.018	*F*_(3,22.64)_ = 3.62; *p* = 0.014
**Altered Mainly by Diet**								
Dubosiella	*F*_(1,1.99)_ = 7.05; *p* = 0.008	ns	*F*_(1,2.63)_ = 35.25; *p* = 2.89 × 10^−9^	ns	ns	ns	*F*_(3,24.33)_ = 3.33; *p* = 0.024	ns
Muribaculaceae	ns	ns	ns	ns	ns	*F*_(3,27.40)_ = 4.02; *p* = 0.008	ns	ns
Lachnospiraceae_NK4A136 group	*F*_(1,1.91)_ = 5.81; *p* = 0.016	ns	*F*_(1,2.66)_ = 28.33; *p* = 1.02 × 10^−7^	ns	ns	*F*_(3,29.80)_ = 3.94; *p* = 0.011	*F*_(3,29.80)_ = 5.09; *p* = 0.003	ns
Lactococcus	*F*_(1,1.89)_ = 14.57; *p* = 0.00013	ns	*F*_(1,2.54)_ = 104.91; *p* = 1.28 × 10^−24^	ns	ns	ns	*F*_(3,34.30)_ = 8.01; *p* = 0.00008	ns
Bifidobacterium	ns	ns	*F*_(1,2.52)_ = 8.36; *p* = 0.004	*F*_(1,2.52)_ = 6.36; *p* = 0.012	ns	ns	ns	ns

**Table 2:** Terminal endpoint physiological and hematology data. The effects of diet and 3g are listed, but there were no significant interaction effects and thus they are not listed in the table. For reference, average values for each parameter (*n* = 40) are listed in the table on the right.

	Diet	3g	Mean ± SEM (Units)
Organ Weights/Body Weight			
Liver	ns	ns	0.043 ± 0.0007 (g)
Lungs (Figure 9A)	ns	F_(1,36)_ = 4.62; ***p* = 0.039**	0.009 ± 0.0006 (g)
Heart	ns	ns	0.007± 0.0004 (g)
Spleen	ns	ns	0.0035 ± 0.0003 (g)
**[Cort] & [Glucose] & Heart Rate**			
Corticosterone	ns	ns	3078 ± 221 (pg/mL)
Glucose	ns	ns	266.8 ± 12.81 (mg/dL)
Heart Rate	ns	ns	528 ± 5.87 (bpm)
**Hematology**			
White Blood Cells	ns	ns	2.10 ± 0.19 (10^3^/μL)
Neutrophil % (Figure 9D)	F_(1,36)_ = 3.13; *p* = 0.086	ns	24.22 ± 1.48 (%)
Lymphocyte % (Figure 9E)	F_(1,36)_ = 3.53; *p* = 0.068	ns	65.94 ± 2.24 (%)
NLR (Figure 9F)	F_(1,36)_ = 4.12; ***p* = 0.049**	ns	0.429 ± 0.05 (a.u.)
Monocyte % (Figure 9G)	F_(1,36)_ = 3.65; *p* = 0.064	ns	3.78 ± 0.45 (%)
Eosinophils % (Figure 9H)	ns	ns	4.28 ± 0.38 (%)
Basophils % (Figure 9I)	F_(1,36)_ = 3.19; *p* = 0.082	ns	1.78 ± 0.33 (%)
Red Blood Cells	ns	ns	7.34 ± 0.27 (10^6^/μL)
Hemoglobin	ns	ns	11.24 ± 0.40 (g/dL)
HCT %	ns	ns	35.47 ± 1.18 (%)
MCV	ns	ns	48.46 ± 0.34 (fL)
MCH (Figure 9B)	F_(1,36)_ = 7.76; ***p* = 0.008**	F_(1,36)_ = 10.47; ***p* = 0.003**	15.34 ± 0.05 (pg)
[MCHC]	ns	ns	31.59 ± 0.18 (g/dL)
RDW-CV % (Figure 9C)	F_(1,36)_ = 8.65; ***p* = 0.006**	F_(1,36)_ = 9.15; ***p* = 0.005**	14.09 ± 0.14 (%)
Platelets	ns	ns	546 ± 36.72 (10^3^/μL)
Mean Platelet Vol.	ns	ns	5.14 ± 0.03 (fL)

Abbreviations: NLR—Neutrophil/Lymphocyte Ratio; HCT—Hematocrit; MCV—Mean Corpuscular Volume; MCH—Mean Corpusuclar Hemoglobin; MCHC—Mean Corpuscular Hematocrit Concentration; RDW-CV—red cell distribution width-coefficient of variation; ns—Not significant; Bold indicates significant *p*-values.

## Data Availability

The original contributions presented in this study are included in the article/Supplementary Material. The sequencing data are publicly available at: https://qiita.ucsd.edu/study/description/16043 (accessed on 12 May 2025). Further inquiries can be directed to the corresponding author.

## Supplementary Materials

The following supporting information can be downloaded at: https://www.mdpi.com/article/10.3390/nu17152417/s1, Figure S1: Low abundance genera altered by prebiotic diet and 3g (part 1); Figure S2: Low abundance genera altered by prebiotic diet and 3g (part 2); Table S1: Statistical analysis for figures S1 and S2; Figure S3: Network and Heatmap analyses; Figure S4: Granulocyte-to-Lymphocyte Ratio.
