# Supplementary material for: A Prebiotic Diet Containing Galactooligosaccharides and Polydextrose Attenuates Hypergravity-Induced Disruptions to the Microbiome in Female Mice
Source: Nutrients. 2025 Jul 24;17(15):2417. doi: 10.3390/nu17152417 (PMC12348649; doi:10.3390/nu17152417)
Supplement: Supplementary file 1 [file nutrients-17-02417-s001.zip › Supplemental_TableS1_Sig_Genera_stats.pdf]

Nonparametric Longitudinal Data (nparLD) Table: ANOVA-Type Statistics (ATSs)

	Weeks -4 - 0 (pre 3g)		Weeks 1 - 4 (post 3g)					
**Lower RA Genera**	**Diet**	**Diet x Time**	**Diet**	**Gforce (3g)**	**Diet x 3g**	**diet x time**	**3g x time**	**diet x 3g x time**
Incertae Sedis (Sup. Fig. 1A)	ns	ns	$F_{(1,2.62)} = 24.28; p = 8.32 \times 10^{-07}$	$F_{(1,2.91)} = 15.85; p = 0.00007$	ns	$F_{(3,30.39)} = 6.13; p = 0.0004$	ns	ns
Weissella (Sup. Fig. 1B)	ns	ns	ns	$F_{(1,2.87)} = 203.57; p = 3.48 \times 10^{-46}$	ns	ns	ns	ns
Lactobacillus (Sup. Fig. 1C)	ns	ns	ns	ns	ns	ns	ns	$F_{(3,29.85)} = 3.95; p = 0.008$
A2 (Sup. Fig. 1D)	ns	$F_{(2,21.44)} = 4.68; p = 0.010$	$F_{(1,2.86)} = 6.39; p = 0.012$	ns	$F_{(1,2.86)} = 11.52; p = 0.0007$	ns	ns	ns
Blautia (Sup. Fig. 1E)	ns	ns	$F_{(1,2.80)} = 43.31; p = 4.67 \times 10^{-11}$	$F_{(1,2.80)} = 8.21; p = 0.004$	ns	ns	ns	ns
Lachnoclostridium (Sup. Fig. 1F)	$F_{(1,1.92)} = 10.86; p = 0.001$	ns	$F_{(1,2.45)} = 3.73; p = 0.053$	$F_{(1,2.45)} = 9.03; p = 0.003$	ns	ns	ns	ns
Erysipelotoclostridium (Sup. Fig. 1G)	$F_{(1,2.71)} = 20.55; p = 5.8 \times 10^{-06}$	ns	$F_{(1,2.71)} = 11.04; p = 0.0009$	$F_{(1,2.71)} = 24.60; p = 7.04 \times 10^{-07}$	$F_{(1,2.71)} = 10.29; p = 0.0067$	ns	ns	ns
Eubacterium_coprostanoligenes_group (Sup. Fig. 1H)	ns	ns	$F_{(1,2.86)} = 18.77; p = 0.00001$	ns	ns	$F_{(3,30.72)} = 4.62; p = 0.004$	ns	ns
Rosburia (Sup. Fig. 1I)	ns	$F_{(2,32.79)} = 3.14; p = 0.044$	$F_{(1,2.24)} = 31.02; p = 2.55 \times 10^{-08}$	ns	ns	ns	ns	ns
Oscillibacter (Sup. Fig. 1J)	ns	ns	$F_{(1,2.47)} = 13.27; p = 0.0003$	$F_{(1,2.47)} = 7.80; p = 0.005$	ns	$F_{(3,34.31)} = 3.50; p = 0.022$	$F_{(3,34.31)} = 3.05; p = 0.037$	ns
GCA-900066575 (Sup. Fig. 1K)	ns	ns	ns	$F_{(1,2.37)} = 4.14; p = 0.042$	ns	$F_{(3,32.38)} = 3.53; p = 0.022$	ns	ns
Colidextribacter (Sup. Fig. 2A)	ns	ns	$F_{(1,2.70)} = 24.77; p = 6.44 \times 10^{-07}$	$F_{(1,2.70)} = 7.98; p = 0.005$	ns	$F_{(3,28.18)} = 6.37; p = 0.0005$	ns	ns
Intestinimonas (Sup. Fig. 2B)	$F_{(1,1.86)} = 4.67; p = 0.031$	$F_{(2,31.07)} = 4.93; p = 0.009$	$F_{(1,2.37)} = 36.95; p = 1.21 \times 10^{-09}$	$F_{(1,2.37)} = 9.78; p = 0.002$	ns	ns	$F_{(3,32.38)} = 4.15; p = 0.011$	ns
Clostridia_vadinBB60_group (Sup. Fig. 2C)	ns	ns	ns	ns	$F_{(1,2.68)} = 4.48; p = 0.034$	ns	ns	ns
Clostridia_UCG-014 (Sup. Fig. 2D)	ns	ns	ns	$F_{(1,2.76)} = 5.68; p = 0.017$	ns	$F_{(3,32.13)} = 3.14; p = 0.028$	ns	ns
Tuzzerella (Sup. Fig. 2E)	$F_{(1,1.97)} = 24.05; p = 9.38 \times 10^{-07}$	$F_{(2,25.56)} = 7.81; p = 0.0004$	$F_{(1,2.61)} = 85.84; p = 1.96 \times 10^{-20}$	ns	$F_{(1,2.61)} = 6.98; p = 0.008$	ns	ns	ns
Enterococcus (Sup. Fig. 2F)	$F_{(1,1.99)} = 8.07; p = 0.005$	ns	ns	$F_{(1,2.86)} = 4.57; p = 0.033$	ns	ns	ns	ns
Anaerotruncus (Sup. Fig. 2G)	ns	ns	$F_{(1,2.84)} = 4.40; p = 0.036$	$F_{(1,2.84)} = 22.92; p = 0.000002$	ns	ns	ns	$F_{(3,21.00)} = 3.87; p = 0.010$
Marvinbryantia (Sup. Fig. 2H)	ns	ns	$F_{(1,2.31)} = 4.94; p = 0.026$	$F_{(1,2.31)} = 29.86; p = 4.65 \times 10^{-08}$	$F_{(1,2.31)} = 4.44; p = 0.035$	ns	$F_{(3,29.16)} = 3.29; p = 0.031$	$F_{(3,29.16)} = 4.27; p = 0.010$
NK4A214_group (Sup. Fig. 2I)	ns	$F_{(2,31.03)} = 4.04; p = 0.018$	$F_{(1,2.69)} = 5.08; p = 0.024$	ns	$F_{(1,2.69)} = 5.92; p = 0.015$	ns	ns	ns
ASF356 (Sup. Fig. 2J)	ns	ns	ns	ns	ns	ns	ns	ns
Enterorhabdus (Sup. Fig. 2K)	$F_{(1,1.84)} = 14.85; p = 0.0001$	ns	$F_{(1,2.76)} = 25.41; p = 4.6 \times 10^{-07}$	$F_{(1,2.76)} = 39.04; p = 4.1 \times 10^{-10}$	ns	ns	ns	ns
Eubacterium_nodatum_group (Sup. Fig. 2L)	ns	$F_{(2,33.81)} = 3.97; p = 0.20$	$F_{(1,2.62)} = 93.40; p = 4.27 \times 10^{-2}$	ns	$F_{(1,2.62)} = 4.69; p = 0.030$	ns	ns	ns
Defluviitaleaceae_UCG-011 (Sup. Fig. 2M)	$F_{(1,1.96)} = 8.94; p = 0.003$	ns	$F_{(1,2.75)} = 16.77; p = 0.00004$	$F_{(1,2.75)} = 25.16; p = 5.28 \times 10^{-07}$	ns	ns	ns	ns
Lachnospiraceae_UCG-004 (Sup. Fig. 2N)	ns	ns	$F_{(1,2.77)} = 6.53; p = 0.011$	ns	ns	ns	ns	ns
[Eubacterium]_xylanophilum group (Sup. Fig. 2O)	$F_{(1,1.82)} = 6.52; p = 0.010$	ns	$F_{(1,2.99)} = 10.00; p = 0.001$	ns	$F_{(1,2.99)} = 5.31; p = 0.021$	ns	$F_{(3,30.50)} = 4.28; p = 0.005$	ns
