# Supplementary figures and images for: A Prebiotic Diet Containing Galactooligosaccharides and Polydextrose Attenuates Hypergravity-Induced Disruptions to the Microbiome in Female Mice
Source: Nutrients. 2025 Jul 24;17(15):2417. doi: 10.3390/nu17152417 (PMC12348649; doi:10.3390/nu17152417)

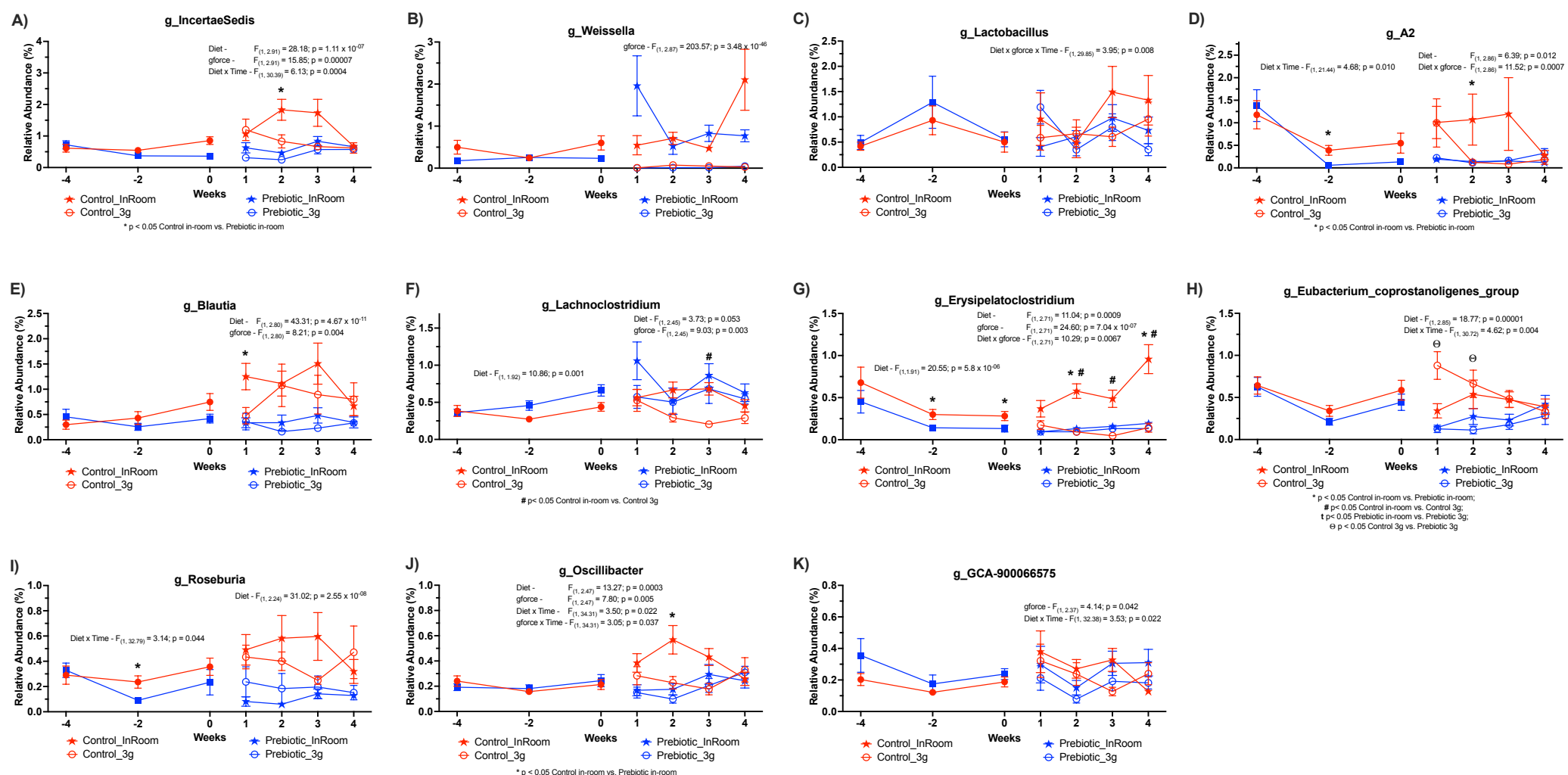

Supplement: Supplementary file 1 [file nutrients-17-02417-s001.zip › SuppFigS1.pdf]

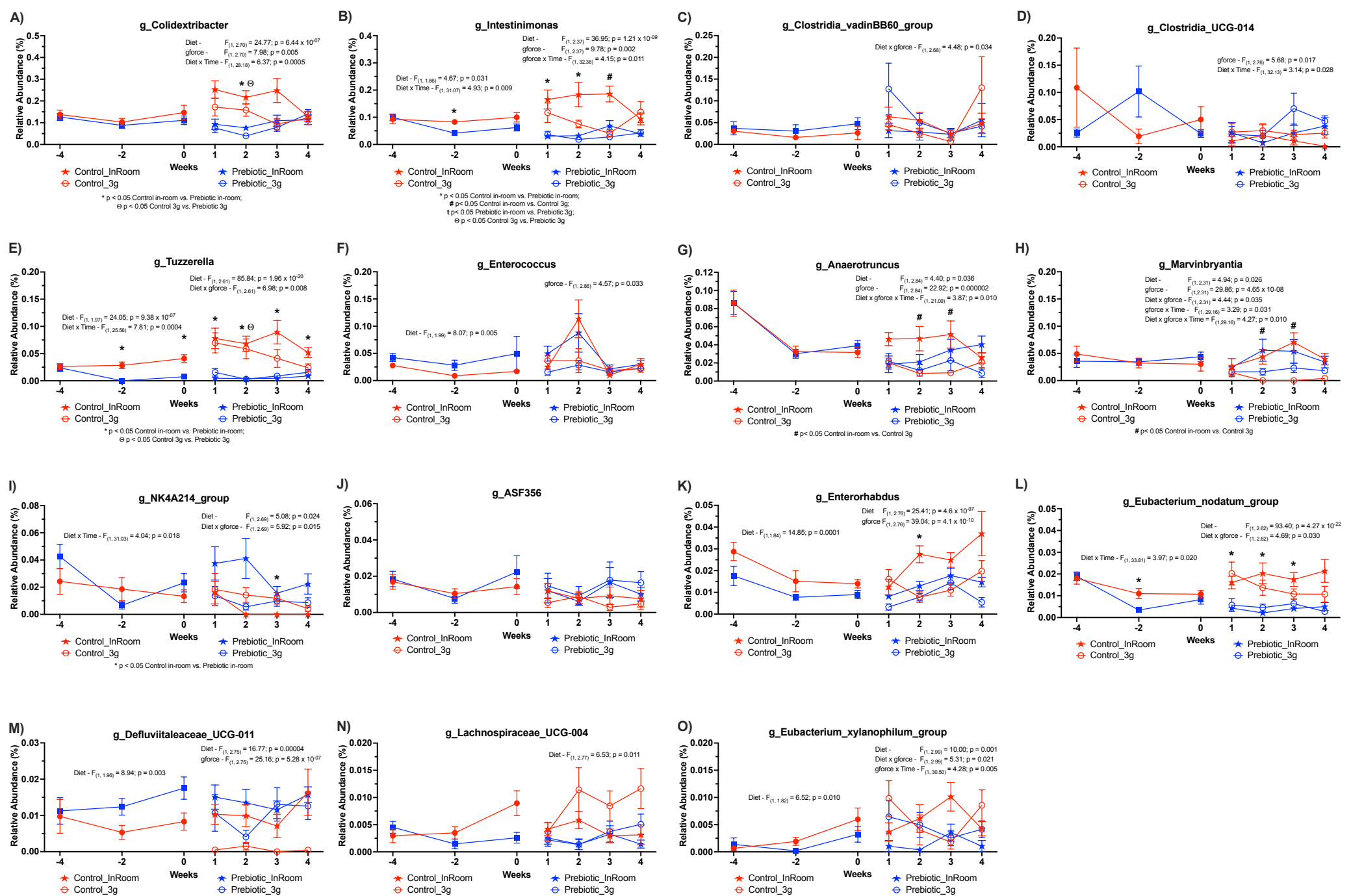

Supplement: Supplementary file 1 [file nutrients-17-02417-s001.zip › SuppFigS2.pdf]

A)

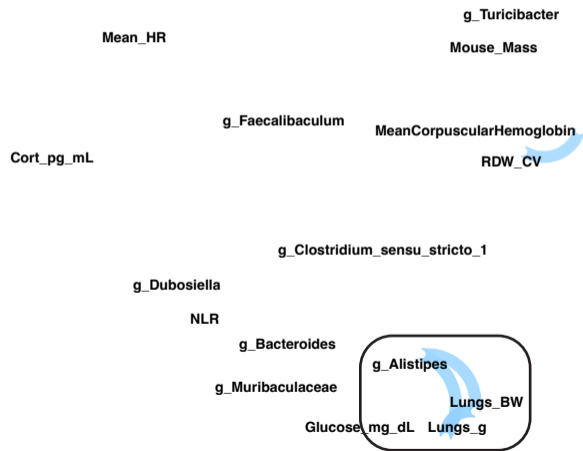

B)

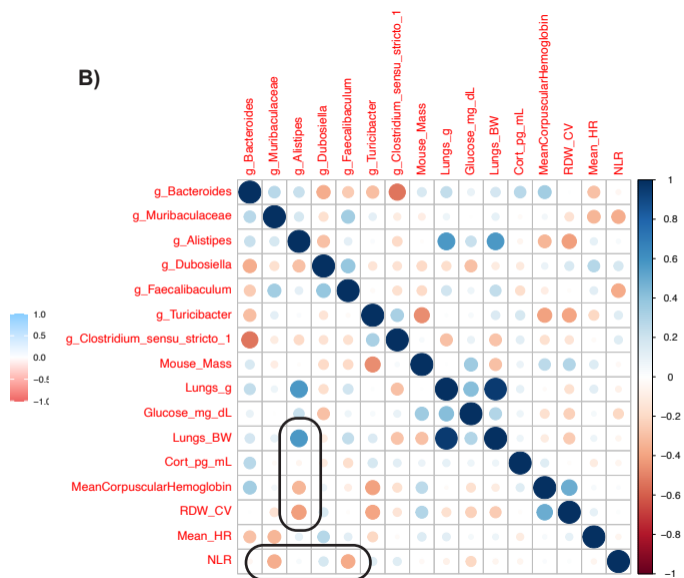

Supplement: Supplementary file 1 [file nutrients-17-02417-s001.zip › SuppFigS3.pdf]

# Granulocyte to Lymphocyte Ratio

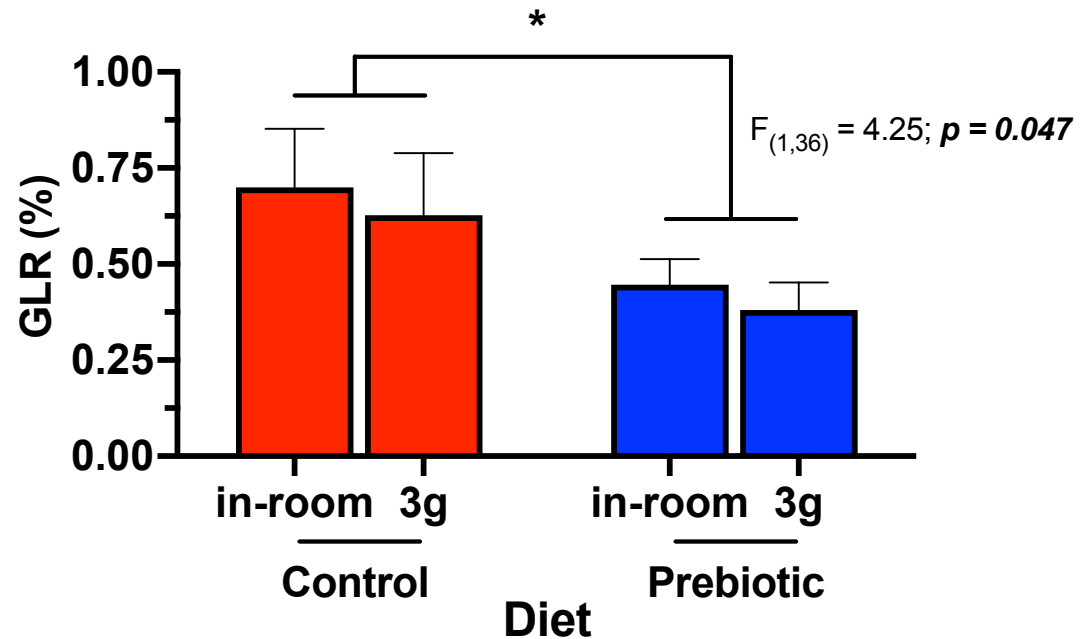

Supplement: Supplementary file 1 [file nutrients-17-02417-s001.zip › SuppFigS4_GLR.pdf]
